# Functional annotation of serine hydrolases in the asexual erythrocytic stage of *Plasmodium falciparum*

**DOI:** 10.1038/s41598-019-54009-0

**Published:** 2019-11-26

**Authors:** Rubayet Elahi, W. Keith Ray, Christie Dapper, Seema Dalal, Richard F. Helm, Michael Klemba

**Affiliations:** Department of Biochemistry, Virginia Tech, Blacksburg, VA 24061 USA

**Keywords:** Biochemistry, Enzymes, Proteomics, Malaria, Chemical tools

## Abstract

Enzymes of the serine hydrolase superfamily are ubiquitous, highly versatile catalysts that mediate a wide variety of metabolic reactions in eukaryotic cells, while also being amenable to selective inhibition. We have employed a fluorophosphonate-based affinity capture probe and mass spectrometry to explore the expression profile and metabolic roles of the 56-member *P. falciparum* serine hydrolase superfamily in the asexual erythrocytic stage of *P. falciparum*. This approach provided a detailed census of active serine hydrolases in the asexual parasite, with identification of 21 active serine hydrolases from α/β hydrolase, patatin, and rhomboid protease families. To gain insight into their functional roles and substrates, the pan-lipase inhibitor isopropyl dodecylfluorophosphonate was employed for competitive activity-based protein profiling, leading to the identification of seven serine hydrolases with potential lipolytic activity. We demonstrated how a chemoproteomic approach can provide clues to the specificity of serine hydrolases by using a panel of neutral lipase inhibitors to identify an enzyme that reacts potently with a covalent monoacylglycerol lipase inhibitor. In combination with existing phenotypic data, our studies define a set of serine hydrolases that likely mediate critical metabolic reactions in asexual parasites and enable rational prioritization of future functional characterization and inhibitor development efforts.

## Introduction

From 2000 to 2017, the number of deaths from malaria was cut in half to 435,000 per year^1^. One important factor contributing to this dramatic decrease was the deployment of artemisinin combination therapies as the frontline treatments for uncomplicated malaria. Unfortunately, parasites resistant to artemisinin and partner drugs such as piperaquine have spread widely across Southeast Asia^2^, leading to concerns that parasite resistance could roll back recent gains. Continuing investment in drug discovery is needed to combat multi-drug resistant parasites.

Identifying and prioritizing druggable targets among the ~5,500 proteins predicted from the genomes of the dominant human malaria species *Plasmodium falciparum*^3^ and *P. vivax*^4^ remains an enormous challenge. A recent saturation mutagenesis study utilizing the *piggybac* transposon has identified 2680 putatively essential coding sequences in the *P. falciparum* genome^5^. Presumably, however, only a small fraction of these will turn out to be feasible targets for anti-malarial drug development. Enzymes that catalyse essential metabolic reactions are attractive in this regard as they frequently possess active site pockets that can serve as targets for high-affinity binding by small molecule inhibitors.

We are interested in exploring the serine hydrolase superfamily as a source of druggable targets in *P. falciparum*. These enzymes have been divided into two functional classes, the serine proteases and the “metabolic” serine hydrolases, many of which are lipases^6,7^. Because many human serine hydrolases are involved in metabolic processes of clinical interest^6,7^, much effort has been directed towards developing chemical biology tools as well as potent and selective inhibitors. The serine hydrolase superfamily includes numerous sub-families (for example, α/β hydrolase, patatin, subtilisin) that have evolved convergently to employ a nucleophilic, active site serine sidechain to catalyse a hydrolytic reaction, typically involving an ester, thioester or amide bond^6^. The catalytic cycle involves the transient formation of a covalent bond between the catalytic serine sidechain and the substrate. The nucleophilicity of the serine hydroxyl group has been leveraged to develop chemical biology reagents for characterizing enzymes of the serine hydrolase superfamily on a proteomic scale. Prominent among these are fluorophosphonate (FP)-based activity-based probes, which react with the active site serine hydroxyl group to generate a stable, covalent enzyme-inhibitor complex. A fluorophore-containing probe permits fluorescence-based visualization of labelled enzymes^8^, whereas appending a (desthio)biotin moiety provides an affinity handle for purification and identification by mass spectrometry^9,10^.

The *P. falciparum* genome encodes 56 proteins that exhibit homology to serine hydrolase superfamily enzymes (Supplementary Table S1). Many of these have unknown physiological roles. To accelerate the discovery and functional characterization of essential malarial enzymes, we have employed a fluorophosphonate probe-based chemoproteomic approach to profile *P. falciparum* serine hydrolases that are expressed during the pathogenic asexual replication cycle. Because little is known about the roles of lipases during asexual development, we employed the pan-lipase inhibitor isopropyl dodecylfluorophosphonate in competitive activity-based protein profiling experiments to identify putative parasite lipases. We then used a small library of neutral lipase inhibitors to further investigate enzyme specificity.

## Results

### Validation of desthiobiotin-fluorophosphonate as an affinity capture probe for *P. falciparum* serine hydrolases

**Figure 1:**
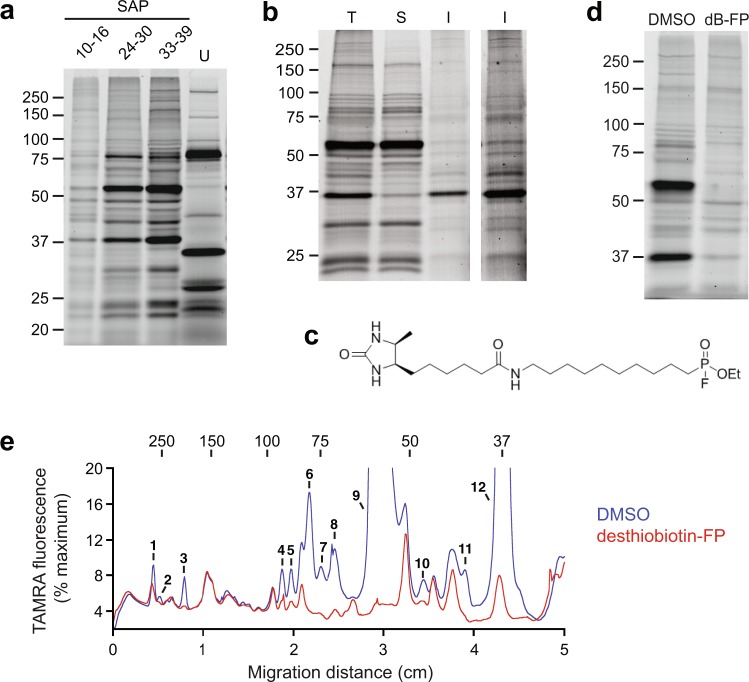
Labelling of saponin-isolated *P. falciparum* serine hydrolases by TAMRA-FP and desthiobiotin-FP. (**a**) TAMRA-FP labelling of lysates prepared from similar numbers of synchronized saponin-isolated parasites (SAP) that were harvested at three time points during the ~42 h asexual replication cycle (times are given as a range in hours post invasion, or hpi) or from uninfected erythrocytes (U). (**b**) Fractionation of TAMRA-FP labelled lysate of saponin-isolated schizont stage parasites. T, total lysate; S, soluble fraction; I, insoluble fraction. The right-most lane shows the insoluble fraction at a higher contrast level. (**c**) Structure of the affinity capture probe desthiobiotin-FP. (**d**) Effect of pre-incubation with 2 µM desthiobiotin-FP (dB-FP) on TAMRA-FP labelling of a lysate of saponin-isolated schizonts. (**e**) Fluorescence profiles of lanes in **d** expressed as a percentage of the maximum signal in the DMSO sample. Peaks with reduced fluorescence intensity in the dB-FP profile are numbered in order from higher to lower molecular mass. In **a**, **b**, **d** and **e**, sizes of molecular markers are indicated in kDa.

We have previously shown that TAMRA-fluorophosphonate (TAMRA-FP) is an effective probe for labelling serine hydrolases in lysates of intraerythrocytic asexual *P. falciparum* parasites that have been isolated with the sterol glycoside saponin^11^. Saponin permeabilizes the erythrocyte and parasitophorous vacuole (PV) membranes, allowing separation of parasites from soluble erythrocyte and PV proteins. Because the erythrocyte and PV membranes remain intact, proteins associated with these membranes co-purify with parasites^12^. To guide our proteomic studies, we assessed the TAMRA-FP labelling pattern at three points across asexual development (Fig. 1a). Both the number of labelled proteins as well as the fluorescence intensity of many of the labelled proteins increased as development progressed. The labelling patterns observed in saponin-isolated parasites were very different from that of the host erythrocyte (Fig. 1a), which suggests that most of the labelled proteins are of parasite origin. We therefore used schizont-stage parasites for our proteomic studies in order to capture the greatest diversity of serine hydrolase expression and to enable the identification of hydrolases that may have roles in daughter merozoite egress and invasion. We then fractionated a TAMRA-FP labelled lysate of saponin-isolated schizont parasites by high-speed centrifugation and assessed the distribution of labelled proteins in soluble and insoluble (*i.e*., microsome pellet) fractions. Distinct TAMRA-FP profiles were observed in the two fractions (Fig. 1b), which prompted us to analyse them separately in the proteomic studies described below.

To examine the potential utility of desthiobiotin-FP (Fig. 1c) for affinity purification of parasite serine hydrolases, a competition experiment was conducted in which lysates of saponin-isolated parasites were pre-incubated with 2 µM desthiobiotin-FP or DMSO vehicle prior to TAMRA-FP labelling. Desthiobiotin-FP pre-treatment effected a reduction in the intensity of TAMRA-FP labelling for most of the proteins observed by one-dimensional polyacrylamide gel electrophoresis (Fig. 1d). Quantitation of fluorescence profiles across gel lanes revealed at least 12 species with reduced TAMRA labelling intensities following desthiobiotin-FP pre-treatment (Fig. 1e). There appeared, however, to be a few TAMRA-FP-labelled proteins that were unreactive with desthiobiotin-FP, which suggests that the nature of the tag on the activity-based probe can exert a strong influence on selectivity in some cases.

### Utility of IDFP as a probe for *P. falciparum* lipases

**Figure 2:**
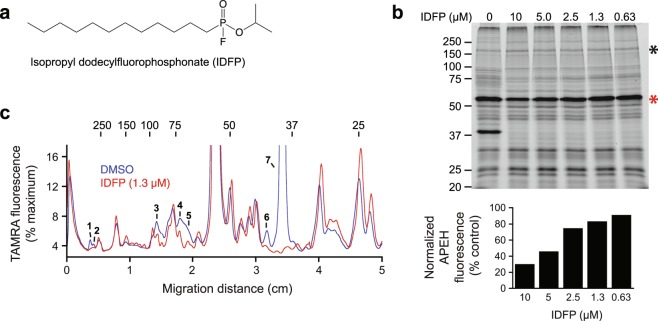
IDFP reacts with a subset of serine hydrolases. (**a**) Structure of IDFP. (**b**) Upper panel: Competition of TAMRA-FP labeling by IDFP (0.63–10 µM). The 55 kDa fragment of human APEH is indicated with a red asterisk and the ~170 kDa band used for normalization of the APEH signal is indicated with a black asterisk. Lower panel: Normalized peak volume for the 55 kDa APEH species *vs*. IDFP concentration. (**c**) Overlay of TAMRA fluorescence profiles for 0 and 1.3 µM IDFP lanes in **b**. Peaks with reduced volume are numbered in order from higher to lower molecular mass. In **b** and **c**, sizes of molecular markers are indicated in kDa.

Isopropyl dodecylfluorophosphonate (IDFP, Fig. 2a) is an isostere of monoacylglycerol that potently inhibits a broad range of mammalian neutral lipases (monoacylglycerol lipase, fatty acid amide hydrolase, KIAA1363) and phospholipases (ABHD3, ABHD6, neuropathy target esterase) with high selectivity over non-lipolytic enzymes such as acetylcholine esterase^13^. To assess the prevalence of *P. falciparum* serine hydrolases with putative lipolytic activity, we conducted a competition experiment in which lysates of saponin-isolated parasites were incubated with a range of IDFP concentrations prior to TAMRA-FP labelling (Fig. 2b). Strong inhibition of labelling was observed for seven species (Fig. 2b,c), which appear to be a subset of the 12 desthiobiotin-FP-reactive species (Fig. 1e). We noted that the non-lipolytic human enzyme acylpeptide hydrolase (APEH, Fig. 2b), which is present in saponin-isolated parasite lysate as a 55 kDa species^11^, was partially inhibited at higher IDFP concentrations. To maximize the selectivity of IDFP for lipolytic enzymes, we sought an IDFP concentration that minimally inhibited APEH. Comparison of normalized APEH peak volumes across the IDFP concentration range revealed that inhibition of APEH was minimal below a concentration of 1.3 µM (Fig. 2b). Thus, a concentration of 1 µM was used in proteomic experiments.

### Proteomic identification of active serine hydrolases in *P. falciparum* schizonts

**Figure 3:**
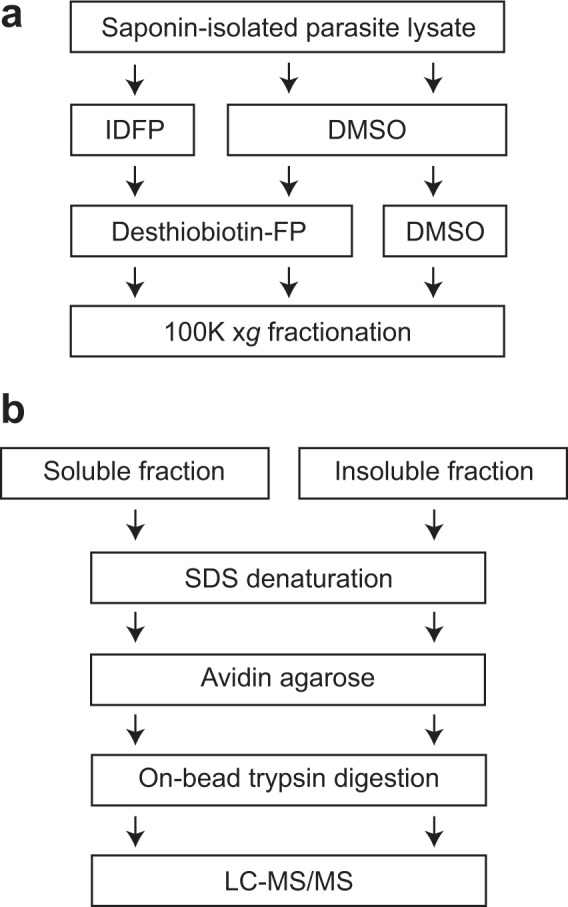
Workflow for desthiobiotin-FP affinity capture proteomic studies. (**a**) Lysate treatment and fractionation. Each biological replicate was divided into three aliquots and treated as indicated. After treatment, each sample was fractionated into soluble and insoluble fractions by high-speed centrifugation. (**b**) Scheme for affinity purification and protein identification from soluble and insoluble fractions.

Desthiobiotin-FP labelling reactions were scaled up for affinity purification and mass spectrometric analysis. To enhance sensitivity for low-abundance microsome-associated proteins (Fig. 1b), 100 K × *g* soluble and insoluble fractions were analysed separately. Three biological replicates were conducted, each originating with an independent saponin-isolated schizont preparation. Each biological replicate was divided into three samples: *i*) desthiobiotin-FP labelled; *ii*) a “no-probe” negative control; *iii*) IDFP pre-incubation followed by desthiobiotin-FP labelling. A schematic overview of the experimental design is provided in Fig. 3. Comparison of *i* and *ii* would yield proteins enriched in a probe-dependent fashion, while comparison of *i* and *iii* would identify putative lipolytic enzymes. Probe-labelled proteins were captured on avidin-agarose beads, subjected to on-bead trypsin digestion and identified by LC-MS/MS.

The relative abundance of individual proteins across samples within the same biological replicate was assessed using two parameters: the numbers of unique peptides and the sum of the areas of the three most abundant unique peptides (the latter is referred to as Top3 protein quantitation, or T3PQ; see Methods). These parameters are reported in Supplementary Tables S2 (soluble fraction) and S3 (insoluble fraction) for all proteins for which at least one unique peptide was observed in the desthiobiotin-FP-labelled samples. To identify desthiobiotin-FP-enriched proteins, the following criteria were applied. Proteins were considered to be enriched with high confidence if, in each biological replicate, they were represented by at least three unique peptides in the desthiobiotin-FP sample and exhibited a ≥10-fold increase in T3PQ in the desthiobiotin-FP *vs*. the no-probe samples. A somewhat less stringent category, denoted as medium-confidence, required at least two unique peptides in two desthiobiotin-FP replicates and at least one peptide in the third replicate, and a ≥10-fold increase in T3PQ in all three replicates. When no peptides were observed in the no-probe control, a minimum peak area based on the detection threshold was used for calculating T3PQ (see Methods). *P. falciparum* proteins with homology to serine hydrolase domains that were observed in our data sets but that did not meet the medium-confidence criteria are reported in Supplementary Table S4.

We have identified 26 desthiobiotin-FP-enriched *P. falciparum* proteins with high or medium confidence, of which 21 are serine hydrolase superfamily homologs (Table 1). Seventeen of these are α/β hydrolase-family enzymes, three are patatin homologs, and one is the rhomboid protease ROM4 (Supplementary Table S1). To assess the efficacy of our enrichment and identification procedure, we consulted the PlasmoDB database^14^ to identify serine hydrolases for which there is untargeted proteomics evidence for expression in the asexual stage and compared this set to our set of desthiobiotin-enriched serine hydrolases (Supplementary Table S1). We found untargeted proteomics evidence for the expression of eleven α/β hydrolase homologs, all but one of which is present in our enriched set. In addition, desthiobiotin-FP affinity capture enabled the identification of an additional seven α/β hydrolases. We also identified all patatin and rhomboid protease homologs for which there is untargeted proteomic evidence for expression in the asexual stage (Supplementary Table S1). In contrast, numerous serine proteases known to be expressed during the asexual stage (subtilisin-like proteases, ClpP, and signal peptidase) were absent from our enriched set. This analysis suggests that desthiobiotin-FP enrichment provides an accurate census of active α/β hydrolase-, patatin- and rhomboid-family members in saponin-isolated schizont lysates but likely misses some serine proteases.

**Table 1:** High- and medium-confidence *P. falciparum* proteins enriched by desthiobiotin-FP affinity purification. Proteins are listed in order of ID. S, soluble; I, insoluble; db-FP, desthiobiotin-FP.

ID PF3D7_	Name/PlasmoDB Annotation^a^	Mass (kDa)^b^	Fraction	Unique Peptides		Confidence	Tn insertion^c^
				db-FP	no probe		
*Serine hydrolase superfamily*							
0209100	Patatin-like phospholipase, putative	78.3	S	1, 3, 2	0, 0, 0	medium	yes
0218600	Patatin-like phospholipase, putative	284	I	18, 16, 40	0, 0, 0	high	yes^d^
0301300	Epoxide hydrolase 1^17^	50.5	I	7, 6, 7	0, 0, 0	high	yes
0321500	Peptidase, putative	126	I	22, 19, 28	0, 0, 0	high	yes
			S	3, 3, 10	0, 0, 1	high	
0403800	Alpha/beta hydrolase, putative	83.4	I	7, 7, 14	0, 0, 0	high	no
0506900	Rhomboid protease ROM4^19^	86.7	I	4, 4, 2	0, 0, 0	medium	no
0629300	Phospholipase^15^	99.2	I	2, 2, 9	0, 0, 0	medium	no
0709700	Prodrug activation and resistance esterase^24^	42.4	I	16, 14, 16	0, 0, 0	high	yes
0728700	Alpha/beta hydrolase, putative	84.8	I	2, 2, 5	0, 0, 0	medium	no
0818600	Plasmodium BEM46-like protein^18^	34.9	I	4, 3, 10	0, 0, 0	high	no
1001600	Exported lipase 2^17^	88.6	S	2, 6, 12	0, 0, 0	medium	no
			I	5, 2, 8	0, 0, 0	medium	
1120400	Alpha/beta hydrolase fold domain containing protein, putative	44.7	I	10, 8, 11	0, 0, 0	high	no
1126600	Steryl ester hydrolase, putative	81.1	I	2, 1, 3	0, 0, 0	medium	yes
1129300	Conserved Plasmodium protein, unknown function	221	I	16, 14, 38	0, 0, 0	high	yes
1134500	Alpha/beta hydrolase, putative	211	I	16, 16, 32	0, 0, 0	high	no
1143000	Alpha/beta hydrolase, putative	44.8	S	7, 9, 14	0, 0, 0	high	no
			I	2, 2, 2	0, 0, 0	medium	
1252600	Esterase, putative	52.9	I	9, 8, 17	0, 0, 0	high	no
			S	1, 2, 7	0, 0, 0	medium	
1328500	Alpha/beta-hydrolase, putative	116	I	7, 7, 13	0, 0, 0	high	yes
			S	1, 2, 2	0, 0, 0	medium	
1358000	Patatin-like phospholipase, putative	238	I	7, 6, 10	0, 0, 0	high	yes
1401300	Epoxide hydrolase 2^17^/proline aminopeptidase^35^	55.2	I	4, 4, 8	0, 0, 0	high	yes
1458300	Conserved Plasmodium protein, unknown function	184	I	10, 9, 19	0, 0, 0	high	yes
*Other proteins*							
0813900	40 S ribosomal protein S16, putative	16.3	I	2, 2, 1	0, 0, 0	medium	no
0914700	Major facilitator superfamily-related transporter, putative	58.4	I	2, 2, 2	0, 0, 0	medium	yes
0930300	Merozoite surface protein 1	196	I	5, 4, 10	0, 0, 1	high	no
1235600	Serine hydroxymethyltransferase	49.8	S	3, 6, 3	0, 0, 0	high	no
1470900	Proteasome subunit beta type-2, putative	22.9	S	1, 4, 7	0, 0, 1	medium	no

^a^When a serine hydrolase has been named in the literature, that name and a reference are provided; otherwise, the PlasmoDB annotation is given.

^b^Predicted molecular mass from PlasmoDB.

^c^From the saturation transposon mutagenesis study by Zhang *et al*.^5^, data obtained from PlasmoDB.

^d^Transposon insertion occurs C-terminal to serine hydrolase domain.

To assess the potential importance of the 21 enriched serine hydrolases for asexual replication in the erythrocyte, we took advantage of a recent large-scale saturation mutagenesis analysis of *P. falciparum*^5^. Coding sequences of 11 of the 21 serine hydrolases have been interrupted by transposon insertions without lethal consequences (Table 1); in all but one of these sequences, the insertion sites were in or N-terminal to the serine hydrolase domain, which strongly suggests that the catalytic activity of these serine hydrolases is not essential for asexual growth. The ten serine hydrolases without reported transposon insertions represent putatively essential proteins for asexual replication of *P. falciparum*. Only four have been previously characterized at a biochemical and/or cellular level in any *Plasmodium* species: PF3D7_0629300, an ortholog of a *P. berghei* phospholipase that contributes to effective sporozoite migration^15^ and merozoite egress from hepatocytes^16^; PF3D7_1001600, termed “exported lipase 2” based on sequence homology and exported into the host erythrocyte^17^; PF3D7_0818600, an ortholog of *P. berghei* BEM46-like protein that influences merozoite development in erythrocytes and sporozoite development in oocysts^18^; and rhomboid protease ROM4, which is responsible for shedding adhesins from the surface of invasive parasite forms^19,20^.

Five medium- and high-confidence *P. falciparum* proteins did not belong to the serine hydrolase superfamily (Table 1). Proteasome subunit β2 has a nucleophilic threonine sidechain that presumably reacted with the probe in a manner analogous to that of serine hydrolases. Serine hydroxymethyltransferase also appears to react with desthiobiotin-FP, as the *Mycobacterium tuberculosis* ortholog has been enriched using the same probe^21^. Three proteins (merozoite surface protein 1, ribosomal subunit S16, and a transporter) have no obvious mode of reaction with desthiobiotin-FP; these likely bound to avidin-agarose beads in a non-specific manner.

Five human serine hydrolases were also identified in our medium- and high-confidence set (Table 2). One of these is acylpeptide hydrolase, which is a soluble erythrocyte enzyme that is internalized by *P. falciparum*^11^. Two other soluble erythrocyte serine hydrolases, prolyl endopeptidase and acetylcholinesterase, may also be internalized. Two serine hydrolases were observed in the insoluble fraction: neuropathy target esterase/PNPLA6 (a phospholipase^22^) and KIAA1363 (also known as AADACL1 and neutral cholesterol ester hydrolase), an enzyme that catalyses the hydrolysis of an acetyl group from a glycerolipid ether^23^. The presence of these membrane proteins can be accounted for by the fact that the erythrocyte plasma membrane remains associated with parasites following saponin treatment^12^.

**Table 2:** High- and medium-confidence human serine hydrolases enriched by desthiobiotin-FP affinity purification. AcMAGE, 2-acetylmonoalkylglycerol ether; S, soluble; I, insoluble; db-FP, desthiobiotin-FP; NC, not calculated due to the absence of peptides in IDFP samples.

ID	Name	Activity	Fraction	Unique Peptides			Ave. T3PQ ratio db-FP:IDFP	Confidence
				db-FP	no probe	IDFP		
P13798	Acylpeptide hydrolase	Peptidase	S	20, 18, 25	1, 0, 7	18, 14, 25	2	high
			I	9, 8, 10	0, 0, 0	5, 4, 8	1	high
P48147	Prolyl endopeptidase	Peptidase	S	2, 8, 16	0, 0, 0	9, 2, 19	2	medium
P22303-2	Acetylcholinesterase	Non-glycerolipid esterase	S	4, 4, 16	0, 0, 0	0, 0, 8	4	high
A0A0A0MTJ9	KIAA1363/AADACL1	AcMAGE hydrolase	I	8, 7, 15	0, 0, 0	4, 1, 6	1	high
Q8IY17-4	Neuropathy target esterase/PNPLA6	Phospholipase	I	26, 18, 35	0, 0, 0	0, 0, 0	NC	high

### Identification of *P. falciparum* serine hydrolases with putative lipolytic activity

The presence of a set of well-characterized erythrocyte serine hydrolases allowed us to test the validity of the notion that IDFP competition with desthiobiotin-FP labelling will selectively deplete serine hydrolases with lipolytic activity. As shown in Table 2, human serine hydrolases with peptidase or non-glycerolipid esterase activities were not substantially depleted by IDFP treatment as judged by the presence of peptides in the IDFP samples along with average desthiobiotin-FP:IDFP T3PQ ratios of less than 10. In contrast, the one enzyme in the set with phospholipase activity (the patatin-containing neuropathy target esterase, or PNPLA6^22^) was so effectively depleted by IDFP competition that no peptides were observed in any biological replicate. KIAA1363/AADACL1 has a lipid-like substrate but was not depleted by IDFP pre-treatment, perhaps because the carboxyl component of the scissile ester bond (acetyl) is small^23^. We conclude from these data that IDFP competition is an effective means of identifying enzymes that act on glycerolipid substrates containing long-chain fatty acyl esters.

The medium- and high-confidence set of 21 *P. falciparum* serine hydrolases was then interrogated for putative lipases by identifying enzymes that either: i) were absent from all IDFP replicates (*i.e*., no peptides were detected); or ii) exhibited average desthiobiotin-FP:IDFP T3PQ ratios greater than 20 in replicates where peptides were present in the IDFP sample. Seven candidate lipases fulfilled these criteria, six of which exhibited a complete or near-complete disappearance of unique peptides from the IDFP-treated samples (Table 3). These six include “prodrug activation and resistance esterase” (PfPARE), an enzyme that can catalyse the hydrolysis of pepstatin esters but has no known physiological substrate^24^, BEM46-like protein^18^ also with no known physiological substrate, and four uncharacterized enzymes. The seventh enzyme, exported lipase 2^17^, exhibited modest decreases in unique peptide numbers and T3PQ ratios, which together suggest partial inhibition by IDFP.

**Table 3:** High- and medium-confidence *P. falciparum* serine hydrolases with putative lipolytic activity. S, soluble; I, insoluble; db-FP, desthiobiotin-FP. NC, not calculated due to the absence of peptides in IDFP samples.

ID PF3D7_	Name/PlasmoDB Annotation	Fraction	Unique Peptides		Ave. T3PQ Ratio
			db-FP	IDFP	db-FP:IDFP
0218600	Patatin-like phospholipase, putative	I	18, 16, 40	0, 0, 0	NC
0709700	Prodrug activation and resistance esterase	I	16, 14, 16	0, 0, 0	NC
0728700	Alpha/beta hydrolase, putative	I	2, 2, 5	0, 0, 0	NC
0818600	Plasmodium BEM46-like protein	I	4, 3, 10	0, 0, 0	NC
1001600	Exported lipase 2	S	2, 6, 12	3, 1, 9	37
		I	5, 2, 8	1, 1, 4	1
1252600	Esterase, putative	I	9, 8, 17	1, 0, 0	1
		S	1, 2, 7	0, 0, 0	NC
1358000	Patatin-like phospholipase, putative	I	7, 6, 10	0, 0, 0	NC

### Inhibitor profiling identifies a serine hydrolase that reacts with monoacylglycerol lipase inhibitors

During the asexual replication cycle, *P. falciparum* produces the neutral lipid triacylglycerol and stores it in lipid droplets^25,26^. Fatty acids are released from these stores during parasite maturation^26^, presumably to provide precursors for phospholipid synthesis. The enzymes responsible for catalysing triacylglycerol hydrolysis in *P. falciparum* are unknown. In human adipocytes, mobilization of fatty acids from neutral lipids is catalysed by a suite of serine hydrolases: adipose triglyceride lipase, hormone sensitive lipase and monoacylglycerol lipase catalyse the hydrolysis of a fatty acyl group from triacylglycerol, diacylglycerol and monoacylglycerol, respectively^27^.

**Figure 4:**
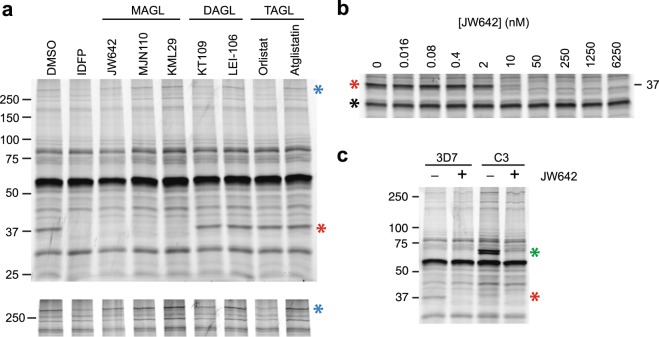
Inhibition of PfPARE by monoacylglycerol lipase inhibitors. (**a**) Competitive activity-based protein profiling of saponin-isolated schizont lysate with a panel of neutral lipase inhibitors (1 µM). MAGL, DAGL and TAGL are mono-, di- and tri-acylglycerol lipase, respectively. A 37 kDa species that reacts with MAGL inhibitors is indicated with a red asterisk. A >250 kDa species that is partially inhibited by DAGL and TAGL inhibitors (KT109 and orlistat, respectively) is indicated with a blue asterisk. The lower panel is the high molecular weight region of the gel image shown at higher contrast. (**b**) Concentration dependence of JW642 inhibition of the 37 kDa species (red asterisk) in saponin-isolated parasite lysate. A labeled protein at ~30 kDa (black asterisk) was used as an internal control for signal normalization. One of three replicates is shown. (**c**) TAMRA-FP labelling profiles of parental parasites (3D7) and a genetically modified line (clone C3) in which the genomic copy of PfPARE (PF3D7_0709700) has been modified to express a C-terminal YFP fusion. Untagged and YFP-tagged PfPARE are indicated with red and green asterisks, respectively. Competitive ABPP was performed with 1 µM JW642. In all panels, the sizes of molecular markers are indicated in kDa.

We speculated that one or more of the putative lipases identified in our set of seven would catalyse the release of fatty acids from parasite triacylglycerol stores. To identify candidate neutral lipases, we conducted competitive activity-based protein profiling (ABPP) experiments with a small library of commercially available, serine hydrolase-directed neutral lipase inhibitors (structures and references are provided in Supplementary Table S5). These inhibitors were employed in gel-based, competitive ABPP experiments at 1 µM concentration in lysates of saponin-isolated schizonts with TAMRA-FP as the activity-based probe (Fig. 4a). This analysis revealed two putative neutral lipases: a 37 kDa species that reacted with all monoacylglycerol lipase (MAGL) inhibitors tested, and a >250 kDa species that was partially inhibited by diacylglycerol lipase (KT109) and pancreatic lipase (orlistat) inhibitors. Both of these proteins were effectively inhibited by 1 µM IDFP (Fig. 4a), and therefore presumably appear in our set of putative lipases (Table 3).

We elected to further characterize the 37 kDa hydrolase with the carbamate JW642, which is a highly potent covalent inhibitor of human MAGL that exhibits exquisite selectivity for this enzyme^28^. Competitive ABPP across a range of JW642 concentrations revealed that it is also a potent inhibitor of the plasmodial enzyme (Fig. 4b, Supplementary Fig. S1), with an IC_50_ value of 2.5 ± 0.6 nM (mean ± SD from three replicates). This value is nearly identical to the 3.7 nM IC_50_ reported for JW642 inhibition of human MAGL using a similar competitive ABPP assay^28^. The remarkable concordance of these values strongly suggests that the *P. falciparum* 37 kDa serine hydrolase has active site features that recapitulate those of human MAGL.

We next sought the identity of the 37 kDa protein. Of the putative lipases identified in Table 3, two have predicted molecular masses close to 37 kDa: PF3D7_0709700 (42 kDa) and PF3D7_0818600 (35 kDa). We had previously generated a transgenic parasite line expressing an endogenous C-terminal fusion of PF3D7_0709700 to yellow fluorescent protein (YFP; Supplementary Fig. S2); therefore, we first tested whether this is the JW642-reactive species by comparing the TAMRA-FP labelling profile of wild-type and genetically modified parasite lines (Fig. 4c). We observed that the 37 kDa protein undergoes a shift to ~65 kDa, which is approximately the size expected for fusion (YFP increases the predicted mass by 29 kDa). Importantly, TAMRA-FP labelling of the YFP fusion was blocked by pre-treatment with 1 µM JW642. Together, these findings indicate that the 37 kDa species is PF3D7_0709700, which has been previously characterized as a prodrug activation and resistance esterase (PfPARE)^24^.

## Discussion

We have demonstrated the utility of desthiobiotin-FP for a proteomic characterization of active serine hydrolases in the late asexual stage of erythrocytic *P. falciparum*. The profile of serine hydrolase enrichment that we observed is consistent with deep coverage of α/β hydrolase, patatin and rhomboid protease families; in contrast, several non-rhomboid serine proteases known to be expressed during the asexual stage were not observed, possibly due to a lack of reactivity with the desthiobiotin-FP probe. By combining our data with the results of a recent transposon mutagenesis study^5^, we have defined a set of ten serine hydrolases that, based on the absence of transposon insertions, may be essential for asexual growth. While the essentiality of each of these needs to be confirmed, we propose that these ten enzymes represent a high-priority set for further investigation of biological roles and potential druggability. Additionally, it is possible that enzymes that appear to be non-essential by transposon mutagenesis have essential functions that are masked by redundancy. It is worth noting that because saponin was used to remove soluble erythrocyte proteins, parasite serine hydrolases that are efficiently exported to the parasitophorous vacuole or erythrocyte cytosol may be absent from our set of enriched proteins.

Competitive activity-based protein profiling (ABPP) with the pan-lipase inhibitor IDFP has revealed seven putative *P. falciparum* lipases, four of which may be important for asexual replication based on the absence of transposon insertions^5^. Because IDFP has been shown to inhibit non-lipolytic enzymes such as acylpeptide hydrolase and carboxypeptidase N^13^, lipase activity will need to be verified experimentally. Notably, physiological substrates are not known for any of the seven enzymes; thus, our findings should help guide future biochemical characterizations. We anticipate that the mass spectrometry-based competitive ABPP approach employed here with IDFP will be broadly applicable to the identification of targets of serine hydrolase inhibitors with anti-malarial activity.

We have identified a serine hydrolase, PfPARE (PF3D7_0709700), that reacts potently in competitive ABPP with the monoacylglycerol lipase inhibitors in a panel of neutral lipase inhibitors with defined specificities. This enzyme is a member of a plasmodial sub-family of α/β hydrolases termed “PST-A”^29^, many of which are subtelomeric (although PfPARE is not). This enzyme has been identified as a pepstatin ester (*i.e*., prodrug) esterase, but its physiological substrate is unknown. Our results suggest that its substrate is a monoacylated glycerolipid, perhaps monoacylglycerol or a lysophospholipid, although this needs to be confirmed through further experiment. Interestingly, despite being one of the most abundant serine hydrolases in lysates of saponin-isolated parasites (Fig. 1b), PfPARE appears not to be required for asexual replication under standard culture conditions. Selection of parasites resistant to a pepstatin ester resulted in a parasite line with a nonsense mutation in PfPARE^24^ and a transposon insertion in the coding sequence has been reported^5^. Consistent with these observations, we find that the addition of 1 µM JW642 to culture medium has no effect on the growth of *P. falciparum*. It is conceivable that PfPARE has a role during growth in the human host that is not apparent under culture conditions.

## Methods

### Parasite culture

*P. falciparum* clone 3D7 was cultured at 1–2% hematocrit in human O^+^ erythrocytes (Interstate Blood Bank) in RPMI 1640 medium supplemented with 27 mM sodium bicarbonate, 11 mM glucose, 0.37 mM hypoxanthine, 10 μg/mL gentamicin and 5 g/L Albumax I (Invitrogen). No ethical approval was necessary for this study as it was deemed by Virginia Tech’s Institutional Review Board that the use of human erythrocytes from anonymous donors did not constitute human subjects research. Cultures were incubated at 37 °C in a 5% CO_2_ incubator. Synchronization was achieved by 5% (v/v) sorbitol treatment^30^.

### Plasmid construction and transfection

The plasmid for the endogenous tagging of PF3D7_0709700 with the yellow fluorescent protein (YFP) allele “citrine”^31^ (Supplementary Fig. S2) was produced as follows. One kilobase of the 3′ end (excluding stop codon) of the PF3D7_0709700 genomic sequence was PCR amplified using Velocity DNA polymerase from *P. falciparum* 3D7 genomic DNA using oligos 302 and 303 and was cloned into the *Xho*I and *Avr*II sites of pPM2CIT2^32^, yielding pPF0709700-CIT2. The fidelity of the PF3D7_0709700 homology region was verified by Sanger sequencing.

To generate transgenic parasites, pPF0709700-CIT2 (100 µg) was transfected into ring-stage *P. falciparum* 3D7 parasites by low-voltage electroporation^33^. WR99210 (10 nM) was added 48 hours after transfection. WR99210-resistant parasites were subjected to two cycles of drug cycling whereby WR99210 was removed for 21 days and then reapplied until resistant parasites reappeared. Genomic DNA was prepared using a DNA blood mini kit (Qiagen). Integration at the PF3D7_0709700 locus was confirmed by PCR using the integration-specific oligo pair 376/800 (Supplementary Fig. S2). The parasite line was cloned by limiting dilution and clone C3 was used for these studies. Sequences of oligonucleotides are provided in Supplementary Table S6.

### Preparation of lysates, TAMRA-FP labelling and competitive ABPP

Synchronized parasite cultures were treated with 0.03% (w/v) saponin in cold Dulbecco’s phosphate-buffered saline pH 7.4 (PBS) for 10 minutes on ice, pelleted by centrifugation at 1940 × *g* at 4 °C for 10 minutes, and washed three times with cold PBS. The number of recovered parasites was determined by counting on a hemocytometer. Parasites were suspended in cold PBS containing the protease inhibitors pepstatin A (5 μM) and *N*-(trans-epoxysuccinyl)-*L*-leucine 4-guanidinobutylamide (E-64, 10 µM) to a density of 5 × 10^8^ parasites/mL. Lysates were generated by three rounds of sonication for 10 seconds at 30% amplitude followed by centrifugation at 17,000 × *g* for 5 minutes at 4 °C to pellet cellular debris. Clarified lysates were snap frozen in liquid N_2_ and stored at –80 °C. Lysates of uninfected erythrocytes were prepared in the same fashion with omission of the saponin treatment.

TAMRA-FP labelling was conducted by adding 0.2 µL of 100 µM TAMRA-FP stock in DMSO (ThermoFisher) to 19.8 µL of parasite or uRBC lysate (~1 × 10^7^ cells/reaction) and incubating for 30 minutes at 30 °C. Reactions were quenched by addition of one volume of 2x reducing SDS-PAGE loading buffer and incubation at 95 °C for 5 minutes. For competitive ABPP experiments, inhibitor was added to a final concentration of 1 µM unless otherwise indicated and reactions were incubated at 30 °C for 20 minutes before addition of TAMRA-FP. Samples were resolved on reducing SDS-polyacrylamide gels. In-gel imaging of TAMRA fluorescence was accomplished using a Typhoon Trio flatbed scanner (GE Healthcare Life Sciences). Fluorescence profiles and peak volume quantitation for labelled proteins were generated using the instrument’s ImageQuant TL v2005 software. Image contrast was adjusted with Adobe Photoshop CC. To calculate the IC_50_ of JW642, the peak volume of the ~37 kDa protein in saponin-isolated parasite lysates was normalized to that of a ~30 kDa protein that was not inhibited by JW642. IC_50_ values were determined by fitting the fractional normalized peak volume (relative to the DMSO control) *vs*. concentration data to a four-parameter sigmoidal curve using KaleidaGraph 4.5 (Synergy Software).

### Desthiobiotin-FP affinity purification

Each desthiobiotin-FP affinity purification was conducted with an amount of lysate corresponding to 2 × 10^9^ saponin-isolated, synchronized late schizont-stage parasites. Lysate preparation was conducted as described in the above section, with the modification that saponin-isolated parasites were suspended at a density of 1.2 × 10^9^/mL. First, clarified lysates were incubated with either 1 µM IDFP (IDFP sample) or 1% DMSO (desthiobiotin-FP and no-probe samples) at 30 °C for 30 minutes with occasional gentle mixing. Next, 2 µM desthiobiotin-FP (ThermoFisher) was added to the IDFP and desthiobiotin-FP samples, and 1% DMSO was added to the no-probe sample. Samples were incubated at 30 °C for 60 minutes with occasional gentle mixing and then were centrifuged at 100 K × *g* for 30 minutes at 4 °C to generate soluble (supernatant) and insoluble (pellet) fractions. Unreacted probe was removed from the soluble fractions by passing through 7 K MWCO Zeba spin desalting columns containing 10 mL resin (ThermoFisher) according the manufacturer’s instructions. The eluates were denatured with 0.5% SDS at 95 °C for 5 minutes, allowed to cool to room temperature, and diluted with PBS to yield an SDS concentration of 0.2%. The insoluble fractions were rinsed twice with 1 mL cold PBS, resuspended in 1 mL cold PBS containing 5 µM pepstatin A and 10 µM E-64, sonicated with 10% amplitude for 10 seconds, and centrifuged at 100 K × *g* for 30 minutes at 4 °C. The washed pellets were resuspended in 0.5 mL of 1% SDS in PBS by gentle sonication, incubated at 95 °C for 10 minutes, allowed to cool to room temperature, and diluted with PBS to 0.2% SDS. Avidin-agarose beads (Pierce #20219, ThermoFisher) were added to the denatured soluble (50 µL packed beads) and insoluble fractions (25 µL packed beads) and were incubated for 3 h at room temperature with constant gentle mixing on an orbital shaker. Beads were washed three times with 1% SDS in PBS and then three times with PBS.

### Mass spectrometry and data analysis

Avidin-bound proteins were subjected to “on-bead” trypsin digestion. Samples were reduced with freshly-prepared 4.5 mM dithiothreitol (DTT) in 50 mM ammonium bicarbonate at 37 °C for 1 h. They were then alkylated by addition of freshly-prepared 10 mM iodoacetamide in 50 mM ammonium bicarbonate and incubation for 1 h at ambient temperature. Unreacted iodoacetamide was disposed by adding 10 mM DTT in 50 mM ammonium bicarbonate. Proteins were digested with 1 µg of mass spec-grade trypsin at 37 °C overnight. The resulting peptide mixtures were purified on a BioPureSPN™ MACRO C18 spin column (Nest Group) according to the manufacturer’s instructions and were vacuum dried. Dried peptides were resuspended in 40 µL of 2% acetonitrile, 0.1% formic acid in water for LC-MS.

Peptide samples were analysed on an Orbitrap Fusion Lumos equipped with an Easy-nLC 1200 UPLC and an Easy Spray nanospray source (ThermoFisher). Peptides were separated on a PepMap RSLC C18 (2 µm particle size, 100 Å pore size, 75 µm inner diameter × 25 cm length; ThermoFisher) reversed phase column. Peptides were eluted with a 2 to 45% solvent B gradient (solvent A: 2% acetonitrile and 0.1% formic acid in water; Solvent B: 80% acetonitrile and 0.1% formic acid in water) over 165 min with a flow rate of 300 nL/min and ion spray voltage of 2500 V. Column temperature was maintained at 55 °C and the ion transfer tube at 275 °C. Data acquisition was made in a data-dependent mode with full MS scans acquired in the Orbitrap mass analyser with a resolution of 120,000 and an m/z scan range of 400–1500 Da in profile, positive ion mode, with charge states of 2–5 included. The automatic gain control (AGC) target value was set at 400,000 with 50 ms maximum injection time. The topmost abundant ions with intensity level over 20,000 counts were selected for higher energy collision-induced dissociation (HCD; CE = 30 ± 3), and the isolation window was 1.4 Da. Dynamic exclusion length was set to 15 s with a 10 ppm tolerance around the selected precursor and its isotopes. Peptides were identified and grouped into proteins using Proteome Discoverer 2.2 (ThermoFisher). All peptides were searched against a ‘target-decoy’ database consisting of the *Plasmodium falciparum* 3D7 reference proteome from UniProt, the UniProt human database (UniProt Release 2017_09), and the corresponding reversed sequences using both the Sequest HT (ThermoFisher) and Mascot (Matrix Science) algorithms. Peptides were also searched against a database of common protein contaminants observed in proteome studies provided with the Proteome Discoverer software. The precursor mass tolerance was set to 10 ppm and the fragment mass tolerance to 0.05 Da. Enzyme specificity was set to trypsin with up to two possible missed cleavages. Carbamidomethylation of cysteines was set as a static modification and acetylation at a peptide N-terminus, oxidation of methionines and pyroglutamate formation on N-terminal glutamines as possible variable modifications. False discovery rate (FDR) was limited to less than 1%. Peptide peaks were matched across runs using the Minora Feature Detector node in combination with the Feature Mapper and Precursor Ions Quantifier nodes within Proteome Discoverer when the peptide was not identified in every run. Each biological replicate was analysed with 2 to 3 technical replicates.

The Top 3 Protein Quantification (T3PQ)^34^ method was employed to quantify the relative abundances of proteins in samples derived from the same biological replicate. The areas of the three most abundant peptides (top 3) for each protein identified were summed and the values were normalized to the value obtained for ovalbumin, a contaminant derived from the avidin-agarose beads. The T3PQ values from technical replicates were averaged. If fewer than 3 unique peptides were identified, the sum of areas (2 peptides) or area (1 peptide) was used for fold change calculation. If no peptides were identified, which occurred frequently in the no-probe control, a minimum detection threshold estimate for T3PQ of 10,000 was used.

## Supplementary information

Supplementary information

Supplementary information

Supplementary information

## Data Availability

The data sets generated during this study are available from the corresponding author upon reasonable request.
