# Supplementary material for: Functional annotation of serine hydrolases in the asexual erythrocytic stage of Plasmodium falciparum
Source: Sci Rep. 2019 Nov 26;9:17532. doi: 10.1038/s41598-019-54009-0 (PMC6879560; doi:10.1038/s41598-019-54009-0)
Supplement: Supplementary file 1 — Supplementary information [file 41598_2019_54009_MOESM1_ESM.pdf]

Supplementary Table S1: *Plasmodium falciparum* coding sequences with homology to serine hydrolase superfamily enzymes, known to us as of July 2019. Sequences are ordered by PlasmoDB ID within each sub-family. Sequences were identified by text searches[1], BLAST protein homology searches, and literature[2-4]. Pseudogenes and serine hydrolase homologs that lack the catalytic serine residue are not included.

ID PF3D7_	PlasmoDB Annotation	Untargeted proteomics evidence for asexual expression[a]	Observed in this study[b]
α/β hydrolase			
0301300	Epoxide hydrolase 1		✓
0321500	Peptidase, putative	✓	✓
0403800	Alpha/beta hydrolase, putative	✓	✓
0629300	Phospholipase	✓	✓
0630100	Conserved Plasmodium protein, unknown function		
0702200	Lysophospholipase, putative		
0709700	Prodrug activation and resistance esterase	✓	✓
0709900	Conserved protein, unknown function		
0728700	Alpha/beta hydrolase, putative	✓	✓
0731800	Alpha/beta hydrolase, putative		
0805000	Alpha/beta hydrolase, putative		
0818600	Plasmodium BEM46-like protein	✓	✓
0823400	Alpha/beta hydrolase, putative		
0826200	Alpha/beta hydrolase, putative		
0936700	Lysophospholipase, putative		
0937200	Lysophospholipase, putative		
1001400	Exported lipase 1	✓	
1001600	Exported lipase 2	✓	✓
1038900	Esterase, putative		
1116100	Serine esterase, putative		
1120400	Alpha/beta hydrolase fold domain … protein, putative		✓
1126600	Steryl ester hydrolase, putative		✓
1129300	Conserved Plasmodium protein, unknown function		✓
1134500	Alpha/beta hydrolase, putative	✓	✓
1143000	Alpha/beta hydrolase, putative	✓	✓
1252600	Esterase, putative		✓
1328500	Alpha/beta hydrolase, putative	✓	✓
1401300	Epoxide hydrolase 2		✓
1401500	Esterase, putative		
1410100	Alpha/beta hydrolase, putative		
1427100	Lipase, putative		
1458300	Conserved Plasmodium protein, unknown function		✓
1476700	Lysophospholipase, putative		
1476800	Lysophospholipase, putative		
Patatin			
0209100	Patatin-like phospholipase, putative	✓	✓
0218600	Patatin-like phospholipase, putative	✓	✓
0814400	Phospholipase DDHD1, putative		
0924000	Patatin-like phospholipase, putative		
1358000	Patatin-like phospholipase, putative	✓	✓

Subtilisin			
0507200	Subtilisin-like protease 3		
0507300	Subtilisin-like ookinete protein SOPT	✓	
0507500	Subtilisin-like protease 1	✓	
1136900	Subtilisin-like protease 2		
Rhomboid			
0506900	Rhomboid protease ROM4	✓	✓
0515100	Rhomboid protease ROM9		
0618600	Rhomboid protease ROM10		
0828000	Rhomboid protease ROM3		
1114100	Rhomboid protease ROM1		
1345200	Rhomboid protease ROM6, putative		
1358300	Rhomboid protease ROM7, putative		
1411200	Rhomboid protease ROM8		
Other serine proteases			
0307400	ClpP	✓	
0807700	Serine protease DegP		
1320400	Type I signal peptidase		
1331300	Signal peptidase complex catalytic subunit SEC11	✓	
1414900	ATP-dependent protease, putative		

[a] ✓ indicates that at least one peptide was observed in any asexual stage (including merozoites) in an untargeted proteomics study as indicated in the "Mass Spec-based Expression Evidence" section of the PlasmoDB entry. Studies focusing on post-translational modifications (*e.g.*, phosphorylation, acetylation) were not included in this analysis.

[b] ✓ indicates medium- or high-confidence enrichment.

Supplementary Table S4: *P. falciparum* serine hydrolases that did not reach the medium-confidence threshold in either fraction. S, soluble; I, insoluble; db-FP, desthiobiotin-FP.

ID PF3D7_	PlasmoDB Annotation	Mass (kDa)[a]	Fraction	Unique Peptides		Tn insertion[b]
				db-FP	no probe	
0731800	Alpha/beta hydrolase, putative	78.4	I	7, 4, 0	0, 0, 0	yes
0805000	Alpha/beta hydrolase, putative	28.5	I	0, 0, 10	0, 0, 0	yes
			S	0, 0, 7	0, 0, 0	
0937200	Lysophospholipase, putative	40.8	I	0, 0, 7	0, 0, 0	yes
			S	0, 0, 2	0, 0, 0	
1116100	Serine esterase, putative	217	I	1, 1, 10	0, 0, 0	yes
			S	0, 0, 2	0, 0, 0	
1476700	Lysophospholipase, putative	41.3	I	0, 0, 7	0, 0, 0	yes
1476800	Lysophospholipase, putative	42.7	I	1, 1, 7	0, 0, 0	no
			S	0, 0, 2	0, 0, 0	

[a] Predicted molecular mass from PlasmoDB.
[b] From saturation mutagenesis study by Zhang *et al*[5], data obtained from PlasmoDB.

Supplementary Table S5: Serine hydrolase-directed neutral lipase inhibitors used for competitive activity-based protein profiling. ABHD6, α/β hydrolase domain-containing protein 6; DAGL, diacylglycerol lipase; MAGL, monoacylglycerol lipase; TAGL, triacylglycerol lipase.

Inhibitor	Structure	Serine hydrolase target(s)
JW642	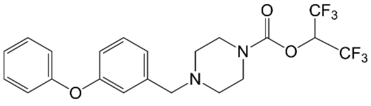	MAGL[6]
MJN110	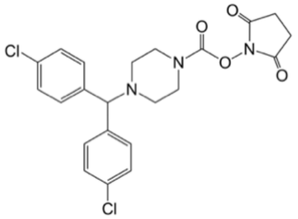	MAGL[7]
KML29	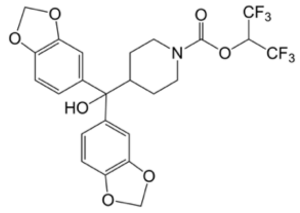	MAGL[6]
KT109	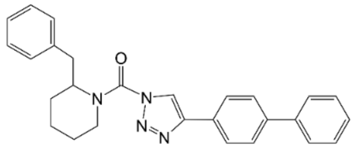	DAGLβ, ABHD6[8]
LEI-106	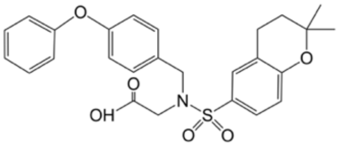	DAGLα, ABHD6[9]
Orlistat	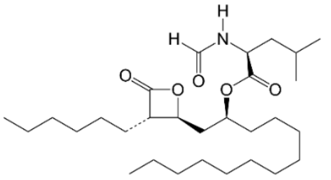	TAGL (pancreatic lipase)[10]
Atglistatin	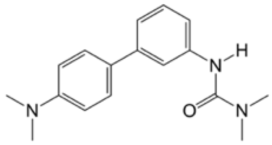	TAGL (adipose triglyceride lipase)[11]

Supplementary Table S6: Oligonucleotides used in this study. RE, restriction enzyme. Underlining indicates RE sites.

Name	Sequence (5' to 3')	RE site	Purpose
302	GCACGCTCGAGTCGAGCAGTAGTCGATTAGATGG	*Xho*I	Forward primer for PCR to generate integration plasmid
303	GCACGCCTAGGTACTTGTTCTTCTTGTTTGGGGGTATG	*Avr*II	Reverse primer for PCR to generate integration plasmid; analytical PCR
376	ATGAAGAGCCAGGGTGGAGGG	none	Forward primer for analytical PCR
800	GTACGGCGGCCGCTTAATGGTGGTGATGGTGGTGTTTGTATAGTTCATCCATGCCATG	*Not*I	Reverse primer for analytical PCR

REFERENCES

1 Bachovchin, D. A. & Cravatt, B. F. The pharmacological landscape and therapeutic potential of serine hydrolases. *Nat. Rev. Drug Discov.* 11, 52-68, doi:10.1038/nrd3620 (2012).

2 Dowse, T. J. & Soldati, D. Rhomboid-like proteins in Apicomplexa: phylogeny and nomenclature. *Trends Parasitol.* 21, 254-258, doi:10.1016/j.pt.2005.04.009 (2005).

3 El Bakkouri, M. *et al.* The Clp chaperones and proteases of the human malaria parasite *Plasmodium falciparum*. *J. Mol. Biol.* 404, 456-477, doi:10.1016/j.jmb.2010.09.051 (2010).

4 Wu, Y., Wang, X., Liu, X. & Wang, Y. Data-mining approaches reveal hidden families of proteases in the genome of malaria parasite. *Genome Res.* 13, 601-616 (2003).

5 Zhang, M. *et al.* Uncovering the essential genes of the human malaria parasite *Plasmodium falciparum* by saturation mutagenesis. *Science* 360, doi:10.1126/science.aap7847 (2018).

6 Chang, J. W. *et al.* Highly selective inhibitors of monoacylglycerol lipase bearing a reactive group that is bioisosteric with endocannabinoid substrates. *Chem. Biol.* 19, 579-588, doi:10.1016/j.chembiol.2012.03.009 (2012).

7 Niphakis, M. J. *et al.* Evaluation of NHS carbamates as a potent and selective class of endocannabinoid hydrolase inhibitors. *ACS Chem. Neurosci.* 4, 1322-1332, doi:10.1021/cn400116z (2013).

8 Hsu, K. L. *et al.* DAGLbeta inhibition perturbs a lipid network involved in macrophage inflammatory responses. *Nat. Chem. Biol.* 8, 999-1007, doi:10.1038/nchembio.1105 (2012).

9 Janssen, F. J. *et al.* Discovery of glycine sulfonamides as dual inhibitors of sn-1-diacylglycerol lipase alpha and alpha/beta-hydrolase domain 6. *J. Med. Chem.* 57, 6610-6622, doi:10.1021/jm500681z (2014).

10 Weibel, E. K., Hadvary, P., Hochuli, E., Kupfer, E. & Lengsfeld, H. Lipstatin, an inhibitor of pancreatic lipase, produced by *Streptomyces toxytricini*. I. Producing organism, fermentation, isolation and biological activity. *J. Antibiot. (Tokyo)* 40, 1081-1085 (1987).

11 Mayer, N. *et al.* Development of small-molecule inhibitors targeting adipose triglyceride lipase. *Nat. Chem. Biol.* 9, 785-787, doi:10.1038/nchembio.1359 (2013).

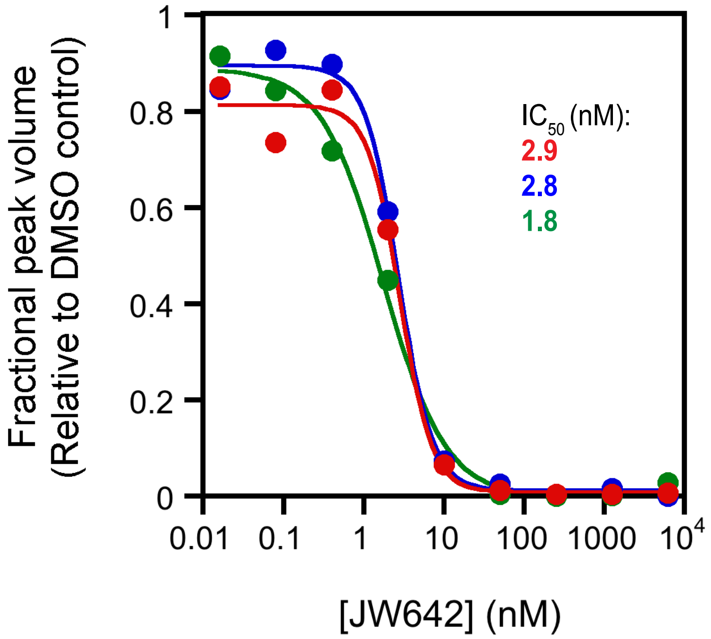

Supplementary Figure S1: Determination of the 50% inhibitory concentration (IC_{50}) for inhibition of PF3D7_0709700 by JW642. Normalized peak volumes for TAMRA-FP-labelled PF3D7_0709700 (Fig. 4b) were expressed as a fraction of the DMSO control and were fit to a four-parameter sigmoidal curve. Results from three replicates are shown.

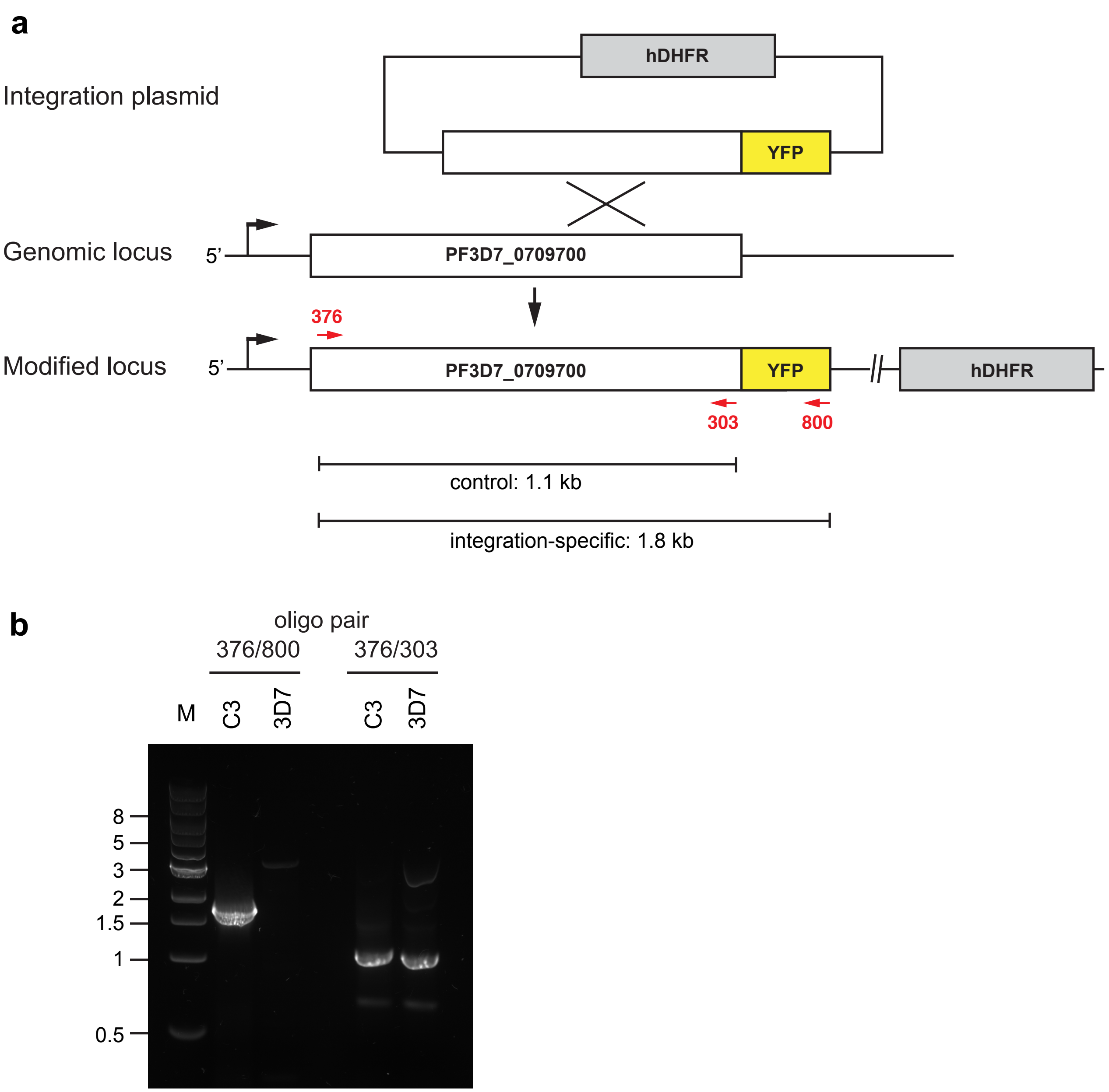

Supplementary Figure S2: Generation of a parasite line modified at the endogenous PF3D7_0709700 locus to express a C-terminal yellow fluorescent protein (YFP) fusion. (**a**) Schematic depiction of the single-crossover homologous recombination event between an integration plasmid and the genomic PF3D7_0709700 coding sequence leading to the generation of a chimera ("modified locus"). Sizes of individual segments are not to scale. The locations and directions of the primers used for PCR are indicated with red arrows. Expected sizes of the control and integration-specific PCR products are shown. (**b**) PCR reactions with equal amounts of genomic DNA from parental parasites (3D7) or from a clonal line (C3) that was obtained following transfection of the integration plasmid and drug cycling. The integration-specific PCR product is observed with clone C3. Sizes of molecular markers (M) are indicated in kilobases.
